# Association of Polymorphisms of Exon 2 of the Growth Hormone Gene with Production Performance in Huoyan Goose

**DOI:** 10.3390/ijms15010670

**Published:** 2014-01-07

**Authors:** Yang Zhang, Zhen Zhu, Qi Xu, Guohong Chen

**Affiliations:** College of Animal Science and Technology, Yangzhou University, Yangzhou 225009, China; E-Mails: zhangyang19860808@126.com (Y.Z.); 18605206923@163.com (Z.Z.); xuqi@yzu.edu.cn (Q.X.)

**Keywords:** multiple comparisons, growth hormone gene, Huoyan goose, production performance, single strand conformation polymorphism

## Abstract

Primers based on the cDNA sequence of the goose growth hormone (GH) gene in GenBank were designed to amplify exon 2 of the GH gene in Huoyan goose. A total of 552 individuals were brooded in one batch and raised in Liaoning and Jiangsu Provinces, China. Single nucleotide polymorphisms (SNPs) of exon 2 in the GH gene were detected by the polymerase chain reaction (single strand conformation polymorphism method). Homozygotes were subsequently cloned, sequenced and analyzed. Two SNP mutations were detected, and 10 genotypes (referred to as AA, BB, CC, DD, AB, AC, AD, BC, BD and CD) were obtained. Allele D was predominant, and the frequencies of the 10 genotypes fit the Hardy-Weinberg equilibrium in the male, female and whole populations according to the chi-square test. Based on SNP types, the 10 genotypes were combined into three main genotypes. Multiple comparisons were carried out between different genotypes and production traits when the geese were 10 weeks old. Some indices of production performance were significantly (*p* < 0.05) associated with the genotype. Particularly, geese with genotype AB or BB were highly productive. Thus, these genotypes may serve as selection markers for production traits in Huoyan geese.

## 1. Introduction

Huoyan goose (*Anser cygnoides*) is a native breed that tolerates coarse feed and cold temperatures, is extremely adaptable, has high egg-laying rates and is weakly broody [1]. It is considered a national treasure by the Chinese goose industry. The egg-laying and reproductive performance of Huoyan geese are outstanding even when considered on a global scale [2]. The inter-population nucleotide divergence of Huoyan goose ranges from 0.211% to 0.272%, notably higher than in other breeds [3]. Thus, studying the genetic characteristics and production performance of Huoyan geese may provide important information for the conservation and utilization of local goose breeds in China.

Growth hormone (GH) is a single polypeptide secreted by eosinophilic granulocytes of the anterior pituitary [4]. It has many physiological functions in animals [5], such as promoting muscle growth [6], bone formation [7] and regulating fat content, all of which are related to and affect the growth and development of animals [8]. In mammals, the GH gene contains five or six exons and four or five introns [9]. The cDNA is typically from 800 to 1200 bp. The length of the signal protein is from 16 amino acid (aa) residues to 27 aa residues, and the length of the mature protein is from 186 aa residues to 191 aa residues [10]. Similarly, the GH gene in poultry also contains five exons and four introns. It is highly polymorphic in a variety of livestock [11–13] and poultry [14,15]. However, much less is known about the polymorphisms in the goose GH gene than in chicken and duck [16–18]. In particular, there has been no systematic study on the relationship between polymorphisms of the GH gene and production performance in Huoyan geese to date. Evidence with several white goose breeds suggests that exon 2 of the GH gene is long, while the other four exons are short, and that all single-nucleotide polymorphisms (SNPs) are in exon 2 [19], making exon 2 the logical target for genetic studies.

In this study, we analyzed polymorphisms in the GH gene of Huoyan geese using the polymerase chain reaction-single strand conformation polymorphism (PCR-SSCP) and established associations between genotypes of exon 2 and some variables of production performance. Some indices of production performance were significantly (*p* < 0.05) associated with the genotype of exon 2. Therefore, polymorphisms in the gene may serve as selection markers for the production performance of Huoyan geese.

## 2. Results and Discussion

### 2.1. Results of Genomic DNA Extraction

The absorption light curve and concentration of the genomic DNA extracted from blood of 10-week-old geese were measured by a NanoDrop ND-1000 concentration analyzer after overnight dissolution in EDTA solution. The curve obtained was smooth, and the optical density (OD260/280) was between 1.8 and 2.0. Thus, the quality of the extracted DNA samples completely satisfies the amplification requirements for PCR-SSCP analysis.

### 2.2. Results of PCR-SSCP Detection

**Figure 1.**
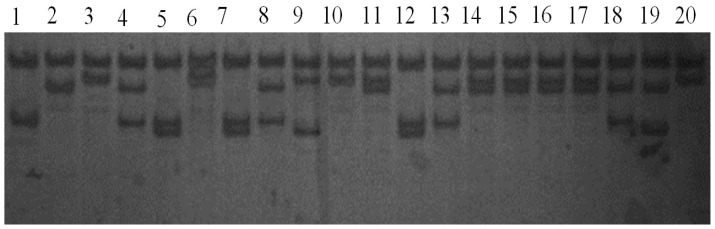
Electrophoresis patterns of exon 2 of the growth hormone (GH) gene of Huoyan goose obtained by polymerase chain reaction-single strand conformation polymorphism (PCR-SSCP). Band 1: genotype BB; Band 2: genotype CC; Bands 3, 6, 10 and 20: genotype DD; Bands 4, 8, 13 and 18: genotype BC; Bands 5, 7 and 12: genotype AB; Band 9: genotype AD; Bands 11, 14, 15, 16 and 17: genotype CD; Band 19: genotype AC.

The amplification product was detected by 1% agarose gel electrophoresis, and the length of the fragment coincided exactly with the target fragment. In addition, no nonspecific banding was amplified. These results indicate that the product can be used directly for PCR-SSCP analysis. Amplification products of exon 2 of the GH gene revealed numerous polymorphisms. There were four homozygous and six heterozygous genotypes, referred to as AA, BB, CC, DD, AB, AC, AD, BC, BD and CD. These results are shown in Figure 1.

### 2.3. Sequencing of Homozygous Genotypes

**Figure 2.**
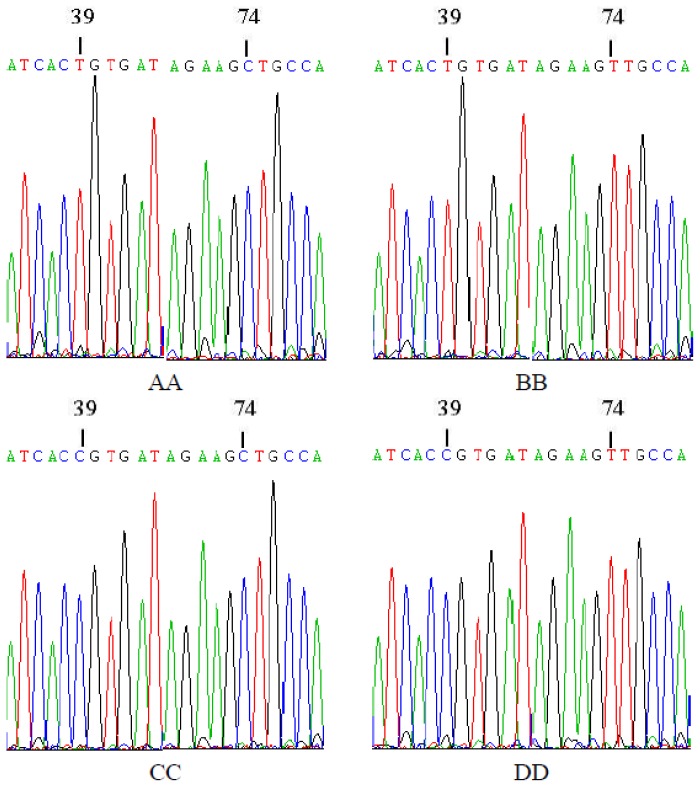
Mutation nucleotide sequence alignment of four homozygous genotypes.

**Figure 3.**
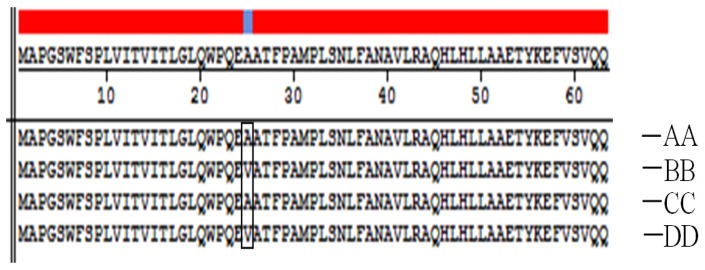
Amino acid sequence comparison of four genotypes. The 25th amino acid residue of the AA and CC genotypes is alanine (**A**), while that of the BB and DD genotypes is valine (**V**).

Four genotypes (AA, BB, CC and DD) were cloned and sequenced by Shanghai Sheng Gong Biological Engineering Technology Service Co., Ltd (Shanghai, China). The sequence alignments produced by the MegAlign program in DNAStar confirmed the findings by Duan [20]. Two single-base mutations were found at the 39th and 74th bp, and the mutations were both T→C transitions. The 39th bp T→C substitution was a silent mutation (the aa sequence was not changed), but the 74th bp mutation resulted in a change in aa 25 from valine→alanine. The sequencing results and aa change are shown in Figures 2 and 3.

### 2.4. Genotypes and Variations in the Allelic Frequencies of Exon 2 of the GH Gene

The genotypes and variations in allelic frequencies determined using the chi-square test are shown in Table 1. Allele D predominated in the male, female and whole populations, with frequencies of 0.3400, 0.3321 and 0.3361, respectively. Both SNP loci conformed to the Hardy-Weinberg equilibrium (male χ^2^ = 0.0600, *p* = 0.498 > 0.05; female χ^2^ = 0.0093, *p* = 0.500 > 0.05; whole population χ^2^ = 0.0170, *p* = 0.498 > 0.05).

### 2.5. Body Weight and Body Traits of Geese with Different Genotypes

Body weights and body traits of 10-week-old geese were assessed. Only relationships among genotypes with mutations at the 74th single base and body traits were analyzed, because silent mutations do not result in changes in the structure of proteins. The 10 genotypes were combined into three main types: the AA, AC and CC genotypes were merged into the AA genotype; the BB, DD and BD genotypes were merged into the BB genotype; and the AB, CD, AD and BC genotypes were merged into the AB genotype. Table 2 shows a comparison of body weight and measurements of body traits for geese with polymorphisms of exon 2 of the GH gene.

Within the male population, there were significant effects of genotype on several body traits, notably on body weight, dive length and sternum length. Geese with genotype AB were heavier than geese with either genotype AA (*p* < 0.01) or BB (*p* < 0.05), and geese of genotype BB were heavier than those with genotype AA (*p* < 0.05). Geese with genotype AB also had longer dive lengths than those with genotypes BB (*p* < 0.01) or AA (*p* < 0.05), but the difference between these two latter populations was not significant. The chest width, chest depth and shank length were also affected significantly by genotypes (*p* < 0.05). Geese with genotype AB had significantly wider chests than those with genotypes AA and BB, but there was no significant difference between the two homozygous genotypes (*p* > 0.05). Birds with genotype AB had longer shanks than those with genotype AA (*p* < 0.05). No differences were observed between birds with genotype BB and those with genotypes AB or AA (*p* > 0.05). Genotype also had no significant effect on inclined body length and shank circumference (*p* > 0.05).

In the female population, genotypes significantly influenced body weight, inclined body length, dive length, chest depth, sternum length (*p* < 0.01) and shank circumference (*p* < 0.05). Birds with genotype BB were significantly heavier (*p* < 0.01) than birds with either genotype AA or AB. Birds with genotype BB had significantly longer sternums (*p* < 0.01) than birds with genotype AA or genotype AB (*p* < 0.05). Birds with genotype AB also had notably longer sternums than those with genotype AA (*p* < 0.05). The shank circumference was also affected significantly by the genotype (*p* < 0.05). Birds with genotype BB had greater shank circumferences than birds with genotype AA (*p* < 0.05). No differences were observed between birds with these two genotypes and genotype AB (*p* > 0.05). Variations among birds of different genotypes in shank length and chest width were not significant (*p* > 0.05).

### 2.6. Carcass Traits of Genotypes

The carcass traits of different genotypes are shown in Table 3. In the male population, geese of genotype AB had significantly heavier glandular and muscular stomachs than those with genotypes AA or BB (*p* < 0.01). No difference was observed between birds with genotypes AA and BB (*p* > 0.05). The carcass weight, semi-eviscerated weight, eviscerated weight and heart weight were significantly affected (*p* < 0.05) by genotype. Carcasses of birds with genotype AB were notably heavier than those of birds with homozygous genotypes (*p* < 0.05), while no difference in carcass weight was observed between birds with the homozygous genotypes (*p* > 0.05). Birds with genotype AB had significantly heavier semi-eviscerated carcasses, eviscerated carcasses and hearts than those with genotype AA (*p* < 0.05). No significant differences in these carcass traits were observed between birds with genotype BB and those with genotypes AA or AB (*p* > 0.05). Breast and leg muscle weights, live weight and abdominal fat weight were not affected by genotypes (*p* > 0.05).

In the female population, the carcass weight and live weight were affected significantly by genotypes. Individuals with genotypes AB and BB had significantly heavier carcasses (*p* < 0.01) than those with genotype AA. Other carcass traits were not significantly affected by genotypes (*p* > 0.05), but with respect to several traits, geese of genotype AB were different from geese with genotypes AB and BB, but the effects were not the same between the sexes (for example, refer to GSW (gland muscular stomach weight) in Table 3). This difference may due to the dominance effect of this locus, and the effect was different in the male and female populations.

### 2.7. Meat Quality of Genotypes

Meat quality of the three genotypes (sexually mixed) is shown in Table 4. The water-holding capacity of the breast and leg muscle were affected significantly by genotype (*p* < 0.05), and the trends were consistent in these two types of muscle. No differences were observed among the three genotypes in terms of pH and tenderness of the muscle. In addition, the water-holding capacity of birds with genotype BB was notably higher than that of birds with genotype AB (*p* < 0.05). No difference between birds with genotype AA and birds of the two other genotypes in terms of water-holding capacity was observed (*p* > 0.05).

### 2.8. Muscle Fiber Traits in Different Genotypes

**Figure 4.**
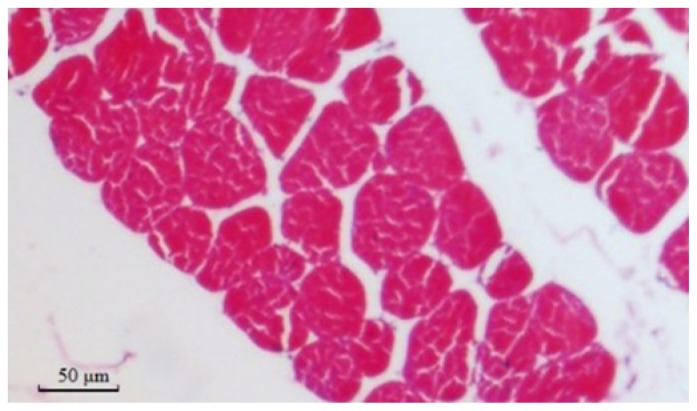
Paraffin section of breast muscle in 10-week-old geese. Muscle fibers were cross-cut. The section was formalin-fixed, paraffin-embedded and counterstained with Mayer’s hematoxylin. In the section, red denotes single muscle fibers and white denotes in-muscle fat. Magnification: 400×.

**Figure 5.**
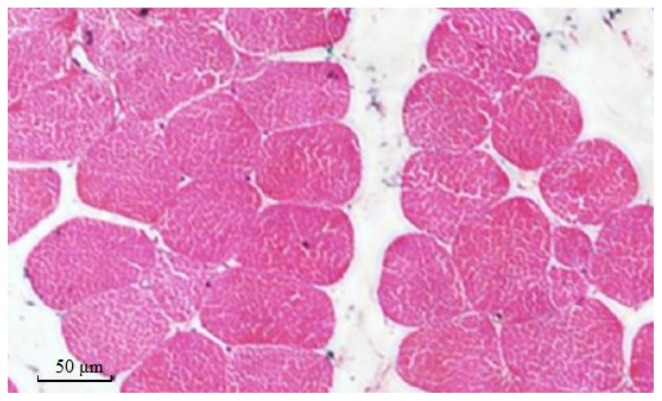
Paraffin section of leg muscle in 10-week-old geese. Muscle fibers were cross-cut. The section was formalin-fixed, paraffin-embedded and counterstained with Mayer’s hematoxylin. In the section, red denotes single muscle fibers and white denotes in-muscle fat. Magnification: 400×.

The muscles were composed primarily of voluntary muscle fibers that could affect taste. The diameter of muscle fibers, an important variable of meat quality, was determined. Hematoxylin and eosin (HE)-stained sections and measurements of breast and leg muscle fibers are shown in Figures 4 and 5 and Table 5. All of the muscle fiber diameters of the male birds were larger than those of the female birds, but no differences were observed among the three genotypes (*p* > 0.05). Mutations of exon 2 of the GH in Huoyan goose affects the diameter of muscle fibers; the effects are limited. Thus, it seems that mutations of exon 2 of the GH gene cannot serve as reliable molecular markers for screening the muscle fiber traits of Huoyan geese.

Other researchers have observed genotypic effects on production traits [21–24]. In our study, two SNP mutations were detected in exon 2 of the GH gene and 10 genotypes were obtained, consistent with previous studies [20]. In all populations, allele D was the predominant allele. Chi-square tests showed that the frequencies of these genotypes fit the Hardy-Weinberg equilibrium, which suggests that Huoyan goose populations are little affected by artificial selection.

There are some possible sources of variation in our experiments. The two Huoyan populations were raised on separate farms, with different climates and latitudes, but they were raised in a similar manner, with identical nutrition, feeding manner and other husbandry management practices. Nonetheless, the differences that existed may have affected the growth and development of the two populations. Body measurements were carried out by dividing the researchers into groups of three, but measurement sites on the animals could not be observed directly, because of the feathers. In addition, the fixation methods used may have introduced errors to the body measurement data. Finally, in addition to the GH gene, the growth of geese is also affected by other sex hormone genes; this may explain the difference between the sexes.

The primary structure of the growth hormone should not be affected by the 39th bp mutation, because of its position in the codon. Thus, only the production traits of three genotypes were analyzed, which improved the test accuracy, precision and reliability and increased the sample size for each genotype. The results of measurement data show that exon 2 of the GH gene may be either the key nucleotide sequence or chained together with other major genes that control or affect some production traits. Genotype AB in the male population and genotype BB in the female population had the most favorable production traits. These molecular markers may serve as candidates for early selection in Huoyan geese.

## 3. Materials and Methods

### 3.1. Experimental Animals and Blood Collection

Blood samples were randomly collected from 616 Huoyan geese from the National Gene Bank of Waterfowl Resources (Taizhou, China) and the Huoyan goose field of Liaoning Academy of Agricultural Sciences (a total of 552 samples were used: 275 from males, 277 from females; 64 deteriorated during transport or due to other factors). Gaggles were raised under normal conditions, and blood was taken from the wing vein on the 10th week. EDTA (4%) was added to the blood to prevent clotting, and samples were stored at −20 °C until use.

### 3.2. Genomic DNA Extraction and Isolation

A hydroxybenzene-chloroform method was used to extract goose genomic DNA, as described in the Molecular Cloning-A Laboratory Manual (2nd edition) [25]. The concentration of each DNA sample was diluted to 100 ng/μL and preserved at 4 °C after detection using a NanoDrop ND-1000 concentration analyzer (Thermo Fisher Scientific Inc., Waltham, MA, USA).

### 3.3. PCR Primer Design and Reagents

A primer pair was designed according to the genomic sequence of the duck GH gene (GenBank Accession No. AB158760), cDNA sequence of the goose GH gene (GenBank Accession No. AY149895) and Duan’s [20] research to amplify the sequence of exon 2. These primer sequences are as follows:

Forward: 5′-GTCGTGGTTTTCTCCTCTC-3′

Reverse: 5′-GCTGTACACTTACGAACTC-3′

The 10× buffer, dNTPs, Mg^2+^ and rTaq enzyme were provided by Takara Biotechnology Co., Ltd. (Dalian, China). The PBR322 DNA/Msp I Marker was purchased from Shanghai Sheng Gong Biological Engineering Technology Services Co., Ltd. (Shanghai, China). Acrylamide, methylene diacrylamide, *N*,*N*,*N*,*N*′-tetramethylethylenediamine, ammonium persulfate and EDTA were supplied by Beijing Dingguo Biotechnology Co., Ltd. (Beijing, China).

### 3.4. PCR-SSCP Analysis

The PCR products were resolved by SSCP analysis. Many factors affect PCR amplification results; so, in this study, gradients, including the template DNA, Mg^2+^ concentration, TaqE dosage and annealing temperature, were all set to obtain the optimum reaction conditions. Each PCR amplification was carried out in a 20 μL mixture containing 2 μL of 10× buffer, 2.5 μL of 25 mmol/L MgCl_2_, 1.0 μL of 10 mmol/L dNTPs, 1 μL of 10 pmol/μL primer, 0.2 μL of 5 U/μL Taq DNA polymerase and 1.0 μL of 100 ng DNA template. Double-distilled water was added to a final volume of 20 μL. PCR was performed using the following program: 95 °C for 5 min, 35 cycles at 94 °C for 30 s, annealing at 52 °C for 30 s and at 72 °C for 30 s and a final extension at 72 °C for 10 min. PCR products were subjected to electrophoresis on 1% agarose gels using 1× TBE buffer (89 mM Tris, 89 mM boric acid, 2 mM Na_2_EDTA) containing 200 ng/mL ethidium bromide. The amplification results were tested using a Gene Genius Glue imaging system (Synoptics Co., Ltd., Cambridge, UK).

Aliquots of the PCR products (6–8 μL) were mixed with 3–4 μL of the denaturing solution (95% formamide, 25 mM EDTA, 0.025% xylene-cyanole and 0.025% bromophenol blue), denatured at 98 °C for 15 min and then quickly chilled on ice for 5–10 min to obtain single-stranded DNA. Approximately 6–10 μL of this mixture was resolved on 12% polyacrylamide gel (polyacrylamide:bisacrylamide = 49:1) using 1× TBE buffer and run for 12–14 h at 120–150 V. The gel was washed in 10% ethanol for 10 min and then in deionized water for 1 min, followed by incubation for 10 min in 0.1% silver nitrate. Finally, incubation in 1.5% sodium hydroxide, 0.01% sodium borohydride and 0.4% (*v*/*v*) formaldehyde was performed for 6–10 min. The bands on the gel were visualized using a gel imaging system.

### 3.5. Sequencing of Homozygous Genotypes

Samples of four homozygous genotypes showing different bands in the gel were further amplified, examined and purified by 1% agarose gel electrophoresis based on the PCR-SSCP analysis results. The products were then sequenced by Shanghai Sheng Gong Biological Engineering Technology Service Co., Ltd. (Shanghai, China).

### 3.6. Statistical Analyses of Production Performance Measurements

Production performance was calculated by analyzing the variance of body weight, seven body measurement indices (inclined body length, dive length, chest width, chest depth, sternum length, shank length and shank circumference; in centimeters), eight carcass traits (slaughter weight, semi-eviscerated weight, eviscerated weight, leg muscle weight, breast muscle weight, heart weight, liver weight and abdominal fat weight; in grams) [26], as well as three meat quality traits (pH, tenderness and water-holding capacity). The pH was measured by a pH meter (Mettler-Toledo FE2; Mettler-Toledo Inc., Shanghai, China). Tenderness was measured by a tenderness meter (CL-ML3; Tianxing Co., Ltd., Beijing, China), and water-holding capacity was measured by a dilatometer (Nanjing Agricultural University, Nanjing, China) on the 10th week. Measurements were carried out according to specific methods detailed in GB Standards (NY/T 823-2004) [27].

Paraffin sections of fresh breast and leg muscles were prepared and dyed with hematoxylin-eosin (HE). A microscopic imaging system (IX71; Olympus (China) Co., LTD, Shanghai, China) was used to capture typical images. The muscle fiber length to long diameter (*L*) and short diameter (*D*) were acquired after 100 measurements using Image Pro-Plus 6.0 microscopic image analysis software (Olympus (China) co., LTD, Shanghai, China). Diameter and cross-sectional areas were computed according to the following formulas.

(1) $$Diameter=\sqrt{\frac{0.7\times L\times D}{3.14}}$$

(2) $$Area=\frac{3.14\times Dia^{2}}{4}$$

Genotypic and allelic frequencies were statistically analyzed using Microsoft Excel (Microsoft Inc., Redmond, WA, USA) according to the published methods [28,29]. Following the formula below, the Hardy-Weinberg equilibrium of the mutations was analyzed by the chi-square test [30].

(3) $$\chi^{2}=\sum \frac{{\left(O-E\right)}^{2}}{E}$$

In Formula 3, *O* represents the observation number of each genotype and *E* represents the expected number of each genotype, if it is assumed that the genotypic distribution conforms to the Hardy-Weinberg equilibrium.

The relationship between the genotypes of exon 2 of the GH gene and 19 traits relating to production performance were analyzed using ANOVA with a range test in SPSS software (version 13.0; International Business Machines Inc., Chicago, IL, USA). The effect of sex was not considered in the analytic process, because production performance was separately analyzed by sex.

## 4. Conclusions

In our study, two SNP mutations were detected, and 10 genotypes (AA, BB, CC, DD, AB, AC, AD, BC, BD and CD) were obtained from 552 Huoyan geese. Allele D was predominant, and the frequencies of the 10 genotypes fit the Hardy-Weinberg equilibrium in the male, female and whole populations, according to the chi-square test. Some indices of production performance were either extremely significantly affected (*p* < 0.01) or significantly affected (*p* < 0.05) by the exon 2 genotype. Genotypes AB and BB were highly productive and may thus serve as molecular markers for the breeding and selection of Huoyan geese with high production performance.

## Figures and Tables

**Table 1.** Genotypic and allelic frequencies at the 39th and 74th bp of exon 2 of the GH gene (*n* = 552). Part A, Genotypic frequencies; Part B, Allelic frequencies.

Populations (Sample Size)	Male (275)	Female (277)	Total Group (552)
Part A. Genotype			

AA	0.0182 (5)	0.0361 (10)	0.0272 (15)
AB	0.0873 (24)	0.0758 (21)	0.0815 (45)
AC	0.1382 (38)	0.1300 (36)	0.1341 (74)
AD	0.1055 (29)	0.1588 (44)	0.1322 (73)
BB	0.0255 (7)	0.0397 (11)	0.0326 (18)
BC	0.1273 (35)	0.0866 (24)	0.1069 (59)
BD	0.1709 (47)	0.1227 (34)	0.1467 (81)
CC	0.0582 (16)	0.0686 (19)	0.0634 (35)
CD	0.1345 (37)	0.1805 (50)	0.1576 (87)
DD	0.1345 (37)	0.1011 (28)	0.1178 (65)

Part B. Allele			

A	0.1836	0.2184	0.2011
B	0.2182	0.1823	0.2002
C	0.2582	0.2671	0.2627
D	0.3400	0.3321	0.3361
χ^2^	0.0600	0.0093	0.0170

**Table 2.** Body weight and other body traits of genotypes of exon 2 of the GH gene. BW, body weight; BIL, inclined body length; DL, dive length; SC, shank circumference; CW, chest width; CD, chest depth; SL, sternum length; SHL, shank length. Data represent the least-squares mean ± SD.

Traits	Genotypes (sample size)					

	AA (♂, 59)	AB (♂, 125)	BB (♂, 91)	AA (♀, 65)	AB (♀, 139)	BB (♀, 73)
BW (g)	2815.27 ± 321.42 aB	3066.61 ± 320.29 Aa	2951.79 ± 322.01 b	2560.49 ± 280.82 B	2646.65 ± 251.18 B	2761.82 ± 283.31 A
BIL (cm)	29.35 ± 2.14	29.92 ± 1.99	29.51 ± 1.82	27.99 ± 1.83 Bb	28.36 ± 1.72 b	28.84 ± 1.73 Aa
DL (cm)	66.62 ± 3.43 b	67.99 ± 3.44 Aa	66.63 ±3.41 Bb	63.43 ± 3.34 Bb	64.07 ± 3.04 b	65.01 ± 2.61 Aa
SC (cm)	4.63 ± 0.32	4.96 ± 0.67	4.79 ± 0.82	4.48 ± 0.25 b	4.57 ± 0.24 ab	4.58 ± 0.26 a
CW (cm)	10.81 ± 0.83 b	11.10 ± 0.83 a	10.87 ± 0.78 b	10.53 ± 0.83	10.59 ± 0.81	10.57 ± 0.84
CD (cm)	7.13 ± 0.52 b	7.19 ± 0.65 a	7.02 ± 0.54 ab	6.96 ± 0.47 Bb	7.06 ± 0.51 b	7.20 ± 0.48 Aa
SL (cm)	11.92 ± 1.15 Bb	12.46 ± 1.30 Aa	12.10 ± 1.20 b	11.54 ± 1.27 Bb	11.87 ± 0.99 a	12.11 ± 0.79 Aa
SHL (cm)	10.79 ± 0.52 b	10.95 ± 0.45 a	10.85 ± 0.54 ab	10.40 ± 0.55	10.36 ± 0.41	10.47 ± 0.41

a,b Means with different superscripts are significantly different (*p* < 0.05).

A,B Means with different superscripts are significantly different (*p* < 0.01).

♂, male population; ♀, female population.

**Table 3.** Carcass traits of geese of different genotypes of exon 2 of the GH gene. Data represent the least-squares mean ± SD. CW, carcass weight; SEW, semi-eviscerated weight: EW, eviscerated weight; BLW, breast and leg muscle weight; HW, heart weight; LW, live weight; GSW, gland muscular stomach weight; AW, abdominal fat weight.

Traits	Genotypes (sample size)					

	AA (♂, 12)	AB (♂, 38)	BB (♂, 33)	AA (♀, 26)	AB (♀, 39)	BB (♀, 16)
CW (g)	2520.35 ± 81.88 b	2754.11 ± 43.19 a	2591.40 ± 63.50 b	2270.86 ± 46.75 B	2430.19 ± 36.83 A	2518.40 ± 43.32 A
SEW (g)	2222.61 ± 67.64 b	2439.32 ± 40.87 a	2343.92 ± 53.39 ab	2033.60 ± 40.92	2331.60 ± 147.94	2230.59 ± 46.24
EW (g)	1974.14 ± 62.83 b	2163.79 ± 39.57 a	2074.64 ± 48.97 ab	1805.63 ± 37.27	2080.31 ± 147.43	1989.69 ± 43.37
BLW (g)	279.34 ± 13.86	286.16 ± 6.87	285.10 ± 8.78	268.00 ± 10.90	296.37 ± 12.60	287.33 ± 12.61
HW (g)	21.37 ± 0.65 b	23.64 ± 0.48 a	23.19 ± 0.60 ab	20.96 ± 0.83	21.500 ± 54	22.66 ± 0.92
LW (g)	78.99±3.66	82.07 ± 2.29	83.34 ± 3.38	68.13 ± 2.94^B^	77.69 ± 2.18^A^	76.04 ± 3.10 AB
GSW (g)	99.87 ± 2.69 B	115.56 ± 2.53 A	102.17 ± 2.93 B	95.93 ± 3.13	97.18 ± 2.37	102.73 ± 2.88
AW (g)	50.60 ± 6.48	54.24 ± 4.46	60.56 ± 4.65	42.92 ± 4.34	54.90 ± 4.97	39.45 ± 5.94

a,b Means with different superscripts are significantly different (*p* < 0.05).

A,B Means with different superscripts are significantly different (*p* < 0.01).

♂, male population; ♀, female population.

**Table 4.** Meat quality of genotypes of exon 2 of the GH gene. Data are expressed as the least-squares mean ± SE.

Traits		Genotypes (sample size)		

		AA (31)	AB (63)	BB (49)
Breast muscle	pH	5.91 ± 0.19	5.93 ± 0.21	5.88 ± 0.18
	TD	45.36 ± 17.43	45.78 ± 13.68	43.67 ± 21.15
	HW	0.30 ± 0.06 ab	0.26 ± 0.08 b	0.30 ± 0.10 a
Leg muscle	pH	5.99 ± 0.20	6.01 ± 0.25	5.98 ± 0.32
	TD	31.82 ± 9.65	30.04 ± 11.70	29.88 ± 9.33
	HW	0.29 ± 0.05 ab	0.27 ± 0.06 b	0.30 ± 0.06 a

a,b Means with different superscripts are significantly different (*p* < 0.05).

TD, tenderness; HW, water-holding capacity.

**Table 5.** Cross-sectional areas of muscle fibers. BM, breast muscle; LM, leg muscle. ♂, male population; ♀, female population.

Traitsμm^2^	Genotypes (sample size)					

	AA (♂, 12)	AB (♂, 38)	BB (♂, 33)	AA (♀, 26)	AB (♀, 39)	BB (♀, 16)
BM	274.46 ± 52.13	330.56 ± 59.13	310.46 ± 57.43	235.57 ± 49.39	241.56 ± 65.83	236.94 ± 51.83
LM	620.97 ± 146.80	670.05 ± 197.46	650.84 ± 143.73	409.48 ± 75.86	607.14 ± 165.18	478.07 ± 92.54

## References

[1] Min Y.N., Hou S.S., Gao Y.P., Huang W., Liu F.Z. (2007). Effect of dietary crude protein and energy on gosling growth performance and carcass trait. Poult. Sci.

[2] Chen G.H., Wang K.H., Wang J.Y. (2004). Goose breeds. Poultry Genetic Resources in China.

[3] Wang J.W., Qiu X.P., Zeng F.T., Shi X.W., Zhang Y.P. (2005). Genetic differentiation of domestic goose breeds in China. Acta Genet. Sin.

[4] Kato Y., Murakami Y., Sohmiya M., Nishiki M. (2002). Regulation of human growth hormone secretion and its disorders. Jpn. J. Med.

[5] Qian M., Liu S.F., Zhuang Z., Lin M.L., Sun Z.Z., Liu C.L., Ma H., Su Y.Q., Tang Q.S. (2012). Genomic structure, polymorphism and expression analysis of the growth hormone(GH) gene in female and male Half-smooth tongue sole (*Cynoglossus* semilaevis). Gene.

[6] Ohlsson C., Bengtsson B.A., Isaksson O.G.P., Andreassen M.C. (1998). Slootweg, growth hormone and bone. Endocr. Rev.

[7] Millar D.S., Horan M., Chuzhanova A.N., Cooper D.N. (2010). Characterisation of a functional intronic polymorphism in the human growth hormone (GH1) gene. Hum. Genomics.

[8] Gordona D.F., Quickb D.P., Erwinc C.R., Donelsonc J.E., Maurer R.A. (1983). Nucleotide sequence of the bovine growth hormone chromosomal gene. Mol. Cell. Endocrinol.

[9] Mou L.J., Liu N., Zadworny D., Chalifour L., Kuhnlein U. (1995). Presence of an additional PstI fragment in intronhormone-encoding gene. Gene.

[10] Harvey S., Scanes C.G., Daughaday W.H. (1995). Growth Hormone.

[11] Hua G.H., Chen S.L., Yu J.N., Cai K.L., Wua C.J., Li Q.L., Zhang C.Y., Liang A.X., Hana L., Geng L.Y. (2009). Polymorphism of the growth hormone gene and its association with growth traits in Boer goat bucks. Meat Sci.

[12] Balogh O., Kovács K., Kulcsár M., Gáspárdy A., Zsolnai A., Kátai L., Pécsi A., Fésüs L., Butler W.R., Huszenicza G. (2009). AluI polymorphism of the bovine growth hormone (GH) gene, resumption of ovarian cyclicity, milk production and loss of body condition at the onset of lactation in dairy cows. Theriogenology.

[13] Dettori M.L., Rocchigiani A.M., Luridiana S., Mura M.C., Carcangiu V., Pazzola M. (2013). Growth hormone gene variability and its effects on milk traits in primiparous Sarda goats. J. Dairy Res.

[14] Zhao W.M., Qiao N., Wang X.B., Chen Q., Cheng J.H., Xu Q., Chen G.H. (2011). Comparative genomic analysis of growth hormone gene in geese. Anim. Sci. J.

[15] Zhang X.L., Jiang X., Liu Y.P., Du H.R., Zhu Q. (2007). Identification of *Ava* I polymorphisms in the third intron of *GH* gene and their associations with abdominal fat in chickens. Poult. Sci.

[16] Ku H., Ni L., Wei G.S. (1997). DNA polymorphisms in the chicken growth hormone gene: Response to selection for disease resistance and association with egg production. Anim. Genet.

[17] Kansaku N., Zadworny D., Guemene D. (2004). Genomic Cloning of Duck Growth Hormone.

[18] Zhan K., Yang N. (2005). Effects of polymorphism in the coding region of GH gene on serum GH, T3 levels and body weight of ducks.

[19] Dong B., Wang J., Duan X.J., Sun G.B., Zhu S.Y., Li X.F. (2010). Polymorphism of the exons of growth hormone (GH) gene in goose. Jiangsu J. Agric. Sci.

[20] Duan X.J., Dong B., Wang J. (2010). Polymorphism in exon 2 of GH gene in six white geese. Anhui Nongxueyuan Xuebao.

[21] Heidari M., Azari M.A., Hasani S., Khanahmadi A., Zerehdaran S. (2012). Effect of polymorphic variants of GH, Pit-1, and β-LG genes on milk production of Holstein cows. Genetika.

[22] Ding X.Z., Liang C.N., Guo X., Xing C.F., Bao J., Chu M., Pei J., Zhu X.S., Yan P. (2012). A novel single nucleotide polymorphism in exon 7 of LPL gene and its association with carcass traits and visceral fat depositionin yak (Bos grunniens) steers. Mol. Biol. Rep.

[23] Lemamy G.J., Guillaume V., Ndeboko B., Mouecoucou J., Oliver C. (2012). Substance P stimulates Growth Hormone (GH) and GH-Releasing Hormone (GHRH) secretions through tachykinin NK2 receptors in sheep. Peptides.

[24] Chang M.T., Cheng Y.S., Huang M.C. (2012). The SNP genotypes of growth hormone gene associated with reproductive traits in Tsaiya ducks. Reprod. Domest. Anim.

[25] Sambrook J., Russel D.W. (2002). Molecular Cloning: A Laboratory Manual.

[26] Zhou Y., Liu Y.P., Jiang X.S., Du H.R., Li X.C., Zhu Q. (2010). Polymorphism of chicken myocyte-specific enhancer-binding factor 2A gene and its association with chicken carcass traits. Mol. Biol. Rep.

[27] Ministry of Agriculture of the People’s Republic of China (2005). Agricultural industry standard. Poultry Production Terms and Measurement Method. NY/T 823-2004.

[28] Nei M., Roychoudhury A.K. (1974). Sampling variances of heterozygosity and genetic distance. Genetics.

[29] Nei M., Li W.H. (1979). Mathematical model for studying genetic variation in terms of restriction endonucleases. Proc. Natl. Acad. Sci. USA.

[30] Dai J.J., Yang A. (2009). A modified chi-squared goodness-of-fit test. J. Math. Res. Appl.

